# Noninvasive electrophysiological imaging identifies 4D uterine peristalsis patterns in subjects with normal menstrual cycles and patients with endometriosis

**DOI:** 10.21203/rs.3.rs-2432192/v1

**Published:** 2023-03-08

**Authors:** Sicheng Wang, Kelsey Anderson, Stephanie Pizzella, Haonan Xu, Zichao Wen, Yiqi Lin, Yuan Nan, Josephine Lau, Qing Wang, Valerie Ratts, Yong Wang

**Affiliations:** Washington University in St. Louis; Washington University School of Medicine in St. Louis; Washington University School of Medicine in St. Louis; Washington University in St. Louis; Washington University in St. Louis; Washington University in St. Louis; Washington University School of Medicine in St. Louis; Washington University School of Medicine in St. Louis; Washington University in St. Louis; Washington University School of Medicine in St. Louis; Washington University School of Medicine in St. Louis

## Abstract

Throughout the menstrual cycle, spontaneous mild contractions in the inner layer of the uterine smooth muscle cause uterine peristalsis, which plays a critical role in normal menstruation and fertility. Disruptions in peristalsis patterns may occur in women experiencing subfertility, abnormal uterine bleeding, ovulatory dysfunction, endometriosis, and other disorders. However, current tools to measure uterine peristalsis in humans have limitations that hamper their research or clinical utilities. Here, we describe an electrophysiological imaging system to noninvasively quantify the four-dimensional (4D) electrical activation pattern during human uterine peristalsis with high spatial and temporal resolution and coverage. We longitudinally imaged 4968 uterine peristalses in 17 participants with normal gynecologic anatomy and physiology over 34 hours and 679 peristalses in 5 participants with endometriosis over 12.5 hours throughout the menstrual cycle. Our data provide quantitative evidence that uterine peristalsis changes in frequency, direction, duration, magnitude, and power throughout the menstrual cycle and is disrupted in endometriosis patients. Moreover, our data suggest that disrupted uterine peristalsis contributes to excess retrograde menstruation and infertility in patients with endometriosis and potentially contributes to infertility in this cohort.

## Introduction

Human uterine activity changes dynamically across the menstrual cycle. Menses begins when serum concentrations of the hormones progesterone and estrogen drop, signaling the uterus to shed blood and epithelial cells through the cervix. In the proliferative phase, the uterine epithelium grows in thickness to prepare for potential embryo implantation as a follicle develops on one or both ovaries to release an oocyte. During the peri-ovulatory phase, an oocyte is released and travels down the fallopian tube. If unprotected sexual intercourse occurs during this time, fertilization may occur. During the secretory phase, the uterine epithelium continues to thicken in preparation for potential embryo implantation.

Most research on the menstrual cycle has focused on hormones and their effects on the epithelium. However, some evidence indicates that the smooth muscle layer, the myometrium, also contributes to uterine functions by generating slow, low-magnitude, spontaneous contractions, termed uterine peristalsis^1–10^. Unlike labor contractions, in which the entire myometrium produces faster and stronger contractions, uterine peristalsis only involves the inner layer of the myometrium, the stratum subvasculare. Uterine peristalsis, first observed on ultrasound^5^, has been shown to vary in direction and frequency throughout the phases of the menstrual cycle^1^. During menses, peristalsis waves travel from the fundus to the cervix and help expel blood and tissue. Conversely, peristalsis waves travel from the cervix toward the fundus during the peri-ovulatory phase and help transport sperm toward the fallopian tubes.

Several studies have suggested that uterine peristalsis plays an essential role in uterine pathology. Disruptions in uterine peristalsis may occur in women who experience infertility^9^, dysmenorrhea^4^, and endometriosis^11,12^, a painful condition in which cells from the uterine epithelium implant and grow outside of the uterus, commonly in the peritoneal space. In addition to causing chronic pelvic pain, endometriosis may also cause dysmenorrhea, irregular bleeding, and subfertility^13^. Evidence that disrupted uterine peristalsis contributes to endometriosis comes from studies using ultrasound and intrauterine pressure catheters. These studies demonstrated that patients with endometriosis had dysperistalsis and higher uterine tone, and more frequent Cervix-Fundus contractions than normal women^8,14,15^.

Although previous studies provided measurements of uterine peristalsis, the available data have been limited by the capabilities of the four main technologies used to assess uterine peristalsis^1,16^. First, intrauterine pressure catheters are invasive, and a catheter placed inside the uterus could alter peristalsis patterns. Second, transvaginal ultrasound (TVUS)^17–19^ is invasive and is not sensitive enough to identify the site of peristalsis initiation. Additionally, the quality of TVUS measurement depends on the orientation of the ultrasound transducer, making this method highly subjective and operator- and time-dependent^20–25^. Third, hysterosalpingography (HSSG) is a procedure in which X-rays are used to detect a radiographic contrast dye injected into the uterus and fallopian tubes. Although HSSG measures are objective, HSSG cannot be used to measure peristalsis amplitude or frequency, and radiation exposure limits the imaging time. Fourth, cine magnetic resonance imaging (MRI)^26–29^ can be used to detect uterine peristalsis by acquiring sequential images for an extended period of time and playing the MRI frames 12 times faster than the actual speed^26^. However, extended cine MRI is expensive, time-consuming, and operator-dependent, and it cannot reveal the initiation and termination sites of uterine peristalsis. Moreover, all of the above modalities can be uncomfortable for the participant and cannot be used for long-term observation.

We recently developed an electrophysiological imaging system called Electromyometrial Imaging (EMMI)^30–33^ to quantitatively measure the electrical activity underlying uterine contractions during labor. Here, we adapted this system to longitudinally image the 4-dimensional (4D) electrical waves of uterine peristalsis over each phase of the menstrual cycle in healthy, nonpregnant participants with normal menstrual cycles and in participants with endometriosis. With this uterine peristalsis imaging (UPI) system, we can image human uterine peristalsis in a safe, comfortable, and accurate way. UPI can provide precise quantitative electrophysiological evidence that uterine peristalsis changes in frequency, direction, duration, magnitude, and power throughout the menstrual cycle and is disrupted in endometriosis patients.

## Results

### Uterine peristalsis imaging (UPI) system

**Figure 1:**
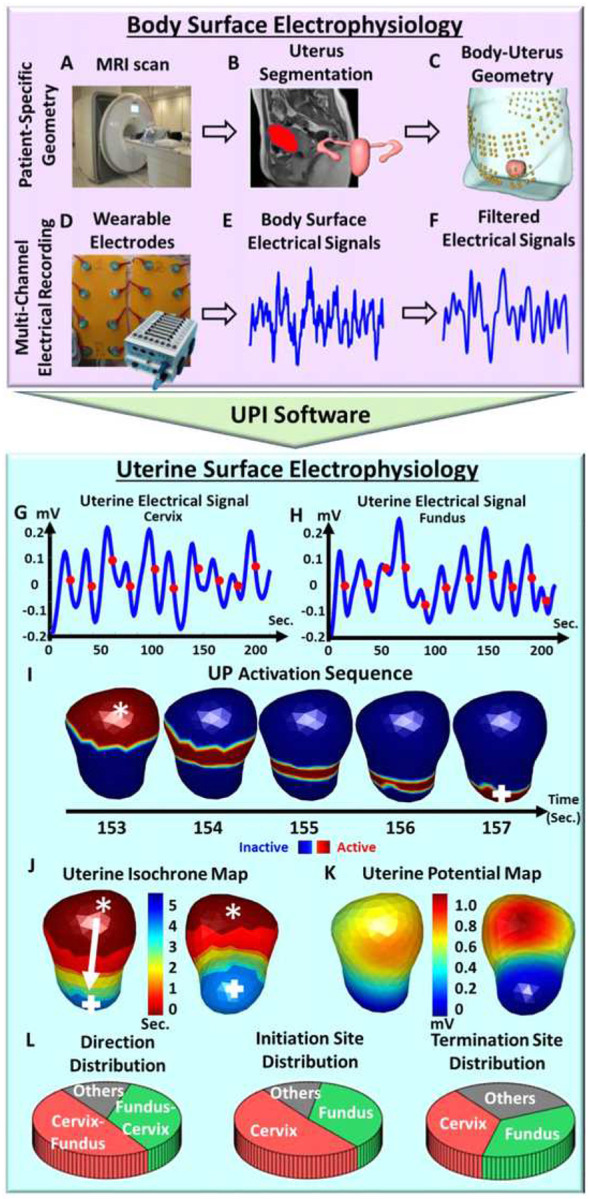
Schematic of uterine peristalsis imaging. (A) A short anatomical MRI determining uterus-body surface geometry. (B) Segmentation of body surface, uterus surface, and fallopian tubes. (C) Patient specific body-uterus geometry. (D) Electrode patches were placed on the patient’s abdomen and back to record body surface electrical signals. (E) Electrical signal measurements on the patient’s body surface. (F) Filtered electrical signals (bandwidth: 0.01–0.1 Hz). (G) Uterine surface electrical signals from one uterine surface point around the fundal region (purple star in J, K, and L). Red dots denote the points of steepest negative slope to represent the activation times during peristalsis cycles. (H) Uterine surface electrical signals from one uterine surface point around the cervical region (green square in J, K, and L). (I) Detailed activation sequence of one complete uterine peristalsis cycle initiated near the fundus and terminated near the cervix. (J) Uterine isochrone maps from the same uterine peristalsis cycle. Warm and cool colors represent early and late activation, respectively. The white arrow depicts the peristalsis propagation direction. (K) One instant uterine potential map from the same uterine peristalsis cycle in I and J represents the potential distribution over the entire 3D uterine surface. (L) Distribution of uterine peristalsis direction (Cervix-Fundus, Fundus-Cervix, others), initiation and termination sites (cervix, fundus, and other areas) analyzed from one electrical mapping. The other three electrophysiological indices, such as magnitude, duration, and power of the uterine peristalsis, were also generated (see details in Materials and Methods)

Our uterine peristalsis imaging (UPI) system is further developed based on the EMMI system and is illustrated in Fig. 1. First, a woman underwent a one-time, fast, anatomical MRI scan (Fig. 1A) to acquire the patient-specific uterus-body surface geometry (Fig. 1B, C), while wearing MRI-compatible fiducial markers around the abdomen and lower back. Second, customized pin-type electrode patches were applied to the same locations on the body surface as the MRI fiducial markers (Fig. 1D). Body surface electrical signals (Fig. 1E) were recorded for 20 minutes, and electrical signals (peristalsis wave signals Fig. 1F) were generated using a band-pass filter (0.01–0.1 Hz)^25,34,35^. Third, UPI software was used to generate electrical signals at each point on the entire 3D uterine surface (Fig. 1G, H). These electrical signals were used to derive activation sequences, uterine potential maps, and uterine isochrone maps (Fig. 1I–K). Finally, the uterine surface data were automatically analyzed to define the peristalsis direction (Cervix-Fundus, Fundus-Cervix, or other), initiation and termination sites (cervix area, fundus area, and other areas), and their distributions (Fig. 1L). Other UPI electrophysiological indices of uterine peristalsis include duration, magnitude, and power of peristalsis waves. See detailed descriptions in the Method section.

### Uterine peristalsis imaging in healthy nonpregnant participants with normal menstrual cycles

**Figure 2:**
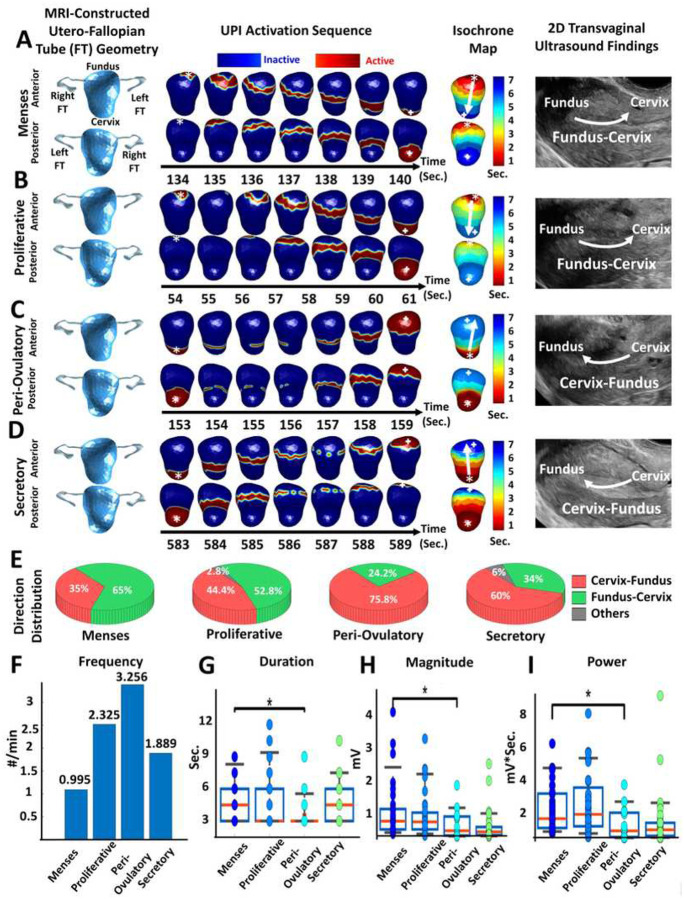
Uterine peristalsis imaging in one participant with regular menstrual cycles during four phases of the menstrual cycle. (A) Dominant Fundus-Cervix uterine peristalsis pattern during the menses phase; (B) Fundus-Cervix pattern during the proliferative phase; (C, D) Dominant Cervix-Fundus uterine peristalsis patterns during the (C) peri-ovulatory phase and (D) secretory phase; (E) Pie charts showing the uterine peristalsis direction distribution in each phase; (F) Bar graph of uterine peristalsis frequency (waves/min); (G,H,I) Boxplots of uterine peristalsis duration (downsampled to 1 Hz, seconds), magnitude (mV), and power (mV*sec) for all peristalsis waves in each phase (each dot represents one uterine peristalsis wave). In the UPI activation sequences and isochrone maps, the white asterisks indicate the peristalsis wave initiation sites, and the white arrows indicate the propagation directions. *P <0.05

We used the UPI system to image uterine peristalsis during each menstrual cycle phase in 17 nonpregnant women with regular menstrual cycles. In total, we imaged 4968 uterine peristalses over 34 hours. In Fig. 2, we present representative uterine peristalsis waves of a 26-year-old participant. During the menses phase, 65% of waves traversed from near the fundus toward the cervix, and 35% traversed from near the cervix toward the fundus (Fig. 2A). During the proliferative phase, 52.8% of waves were Fundus–Cervix and 44.4% were Cervix-Fundus (Fig. 2B). During the ovulatory phase, 75.8% of waves were Cervix-Fundus, and 24.2% were Fundus-Cervix (Fig. 2C). In the secretory phase, 60% of waves were Cervix-Fundus, and 34% were Fundus-Cervix (Fig. 2D). In all cases in which we were able to determine the direction of peristalsis in TVUS images (n = 111), the direction of peristalsis imaged by UPI matched the direction observed by TVUS. Overall, uterine peristalsis waves during menses were significantly longer in duration and had greater magnitude and power than those during the ovulatory phase (Fig. 2F–I).

### Uterine peristalsis imaging in nonpregnant participants with endometriosis

**Figure 3:**
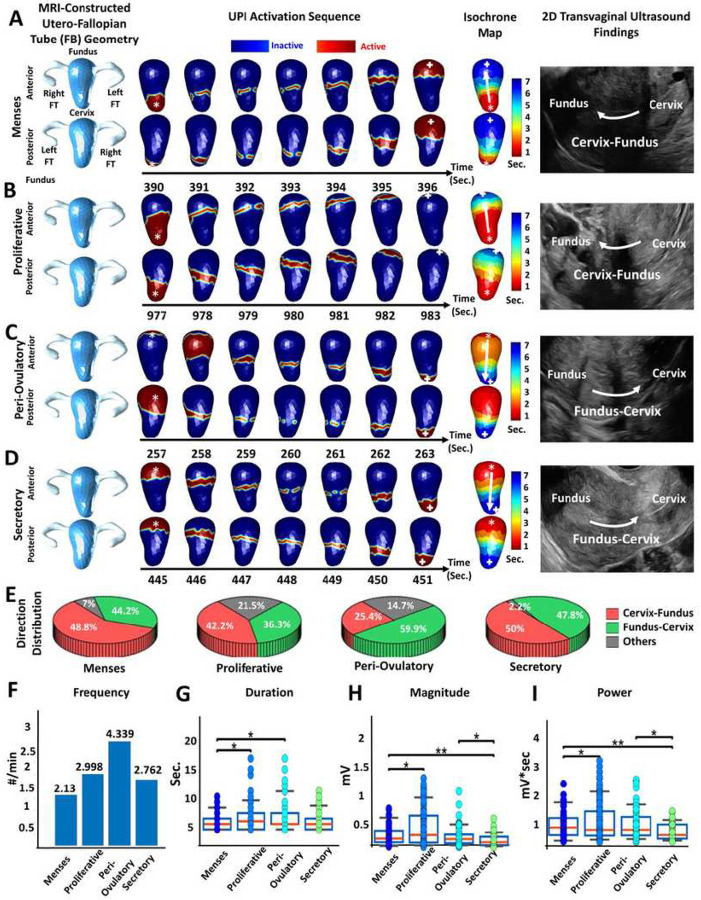
Uterine peristalsis imaging in one participant with surgically confirmed endometriosis during four phases of the menstrual cycle. (A) Dominant Cervix-Fundus uterine peristalsis pattern during the menses phase;(B) Cervix-Fundus uterine peristalsis pattern during the proliferative phase; (C, D) Fundus-Cervix uterine peristalsis pattern during the (C) peri-ovulatory and (D) secretory phases; (E) Pie charts showing the uterine peristalsis direction distribution in each phase; (F) Bar plot of uterine peristalsis frequency (waves/min); (G,H,I) Boxplots of uterine peristalsis duration (downsampled to 1 Hz, seconds), magnitude (mV) and power (mV*sec) for all peristalsis waves in each phase (each dot represents one uterine peristalsis wave). In the UPI activation sequences and isochrone maps, the white asterisks indicate the peristalsis wave initiation sites, and the white arrows indicate the propagation directions. *P <0.05, **P<0.01

We used our UPI system to image uterine peristalsis during each phase of the menstrual cycle in five nonpregnant women with surgically confirmed endometriosis. In total, we imaged 679 peristalses over 12.5 hours throughout the menstrual cycle. In Fig. 3, we present representative uterine peristalsis waves of a 30-year-old participant with endometriosis. During the menses phase (Fig. 3A), 44.2% of waves were Fundus-Cervix, and 48.8% were Cervix-Fundus. During the proliferative phase (Fig. 3B), 36.3% of waves were Fundus-Cervix, and 42.2% were Cervix-Fundus. During the ovulatory phase (Fig. 3C), 59.9% of waves were Cervix-Fundus, and 25.4% were Fundus-Cervix. During the secretory phase (Fig. 3D), 47.8% of waves were Cervix-Fundus, and 50% were Fundus-Cervix. In all cases in which we were able to determine the direction of peristalsis in TVUS images (n = 126), the direction of peristalsis imaged by UPI matched the direction observed by TVUS. Overall, uterine peristalsis waves during menses were significantly shorter in duration than those during the ovulatory phase and had greater magnitude and power than those during the secretory phases (Fig. 3F–I).

### Comparison of uterine peristalsis during the menstrual cycle in healthy participants and endometriosis patients

**Figure 4:**
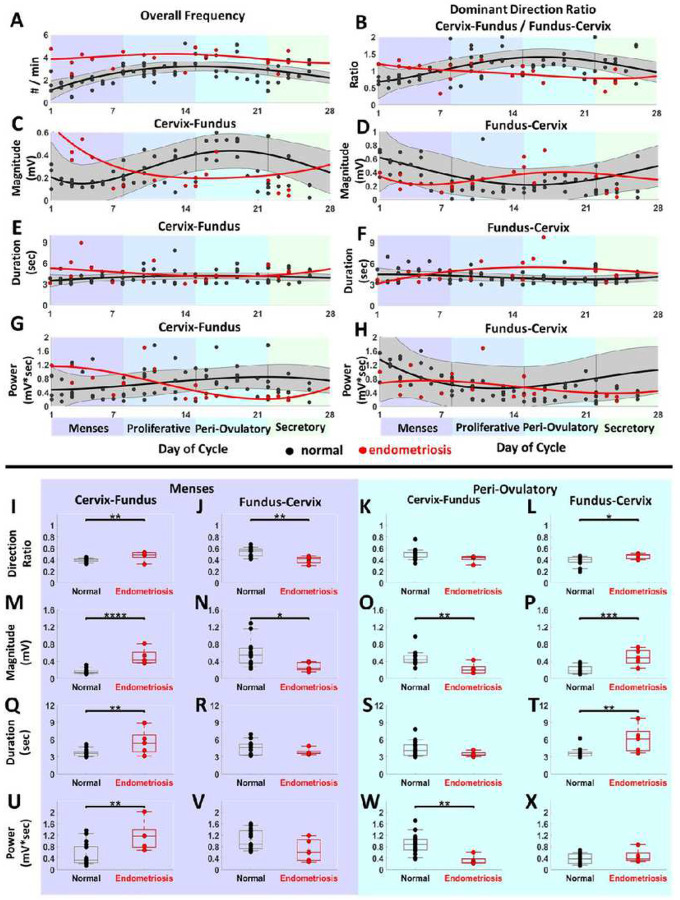
Longitudinal study of uterine peristalsis in normal participants and participants with endometriosis throughout the menstrual cycle. (A-H) Multi-parametric uterine peristalsis quantifications in the standardized 28-day menstrual cycle. Black and red dots represent the average uterine peristalsis measurements of each participant with regular menstrual cycles and endometriosis, respectively. Black curves with grey regions show the confidence regions of fitted multi-parametric uterine peristalsis curves in participants with normal menstrual cycles. Red curves show the fitted multi-parametric uterine peristalsis curves in participants with endometriosis. (I-J, M-N, Q-R, U-V) Group difference analysis of healthy participants and endometriosis patients during the menses phase. The black/red cross in each boxplot shows the median values. (K-L, O-P, S-T, W-X) Group difference analysis of healthy participants and endometriosis patients during the peri-ovulatory phase. N= 17 healthy participants with 4968 uterine peristalsis waves and 5 participants with endometriosis with 679 uterine peristalsis waves. *P<0.05, ** P< 0.01, ***P< 0.001, ****P<0.0001

We next compiled all our data from the healthy and endometriosis participants. The length of each participant’s menstrual cycle was normalized to 28 days. We plotted each participant’s overall frequency and dominant direction ratio (the percentage of Cervix–Fundus peristalsis waves over the percentage of Fundus–Cervix peristalsis waves) (Fig. 4 A–B). We also graphed the average magnitude, duration, and power of peristalsis waves from each participant, with data from the Fundus–Cervix waves plotted separately from the data from Cervix–Fundus waves (Fig. 4 C–H). We observed significant differences in multiple uterine peristalsis indices between healthy participants and those with endometriosis (Fig. 4 I–X). During the menses phase, peristalsis waves were significantly more likely to be Fundus–Cervix in healthy participants than in those with endometriosis (Fig. 4J). The Fundus–Cervix waves were longer (Fig. 4R) and had a higher magnitude (Fig. 4N) in healthy participants than in those with endometriosis. Conversely, the Cervix–Fundus waves were longer duration (Fig. 4Q) and higher magnitude (Fig. 4M) and power (Fig. 3U) in the participants with endometriosis than in the healthy patients. In the peri-ovulatory phase, peristalsis waves were more likely to be Cervix–Fundus in the healthy participants than in the participants with endometriosis (Fig. 4K), and the Cervix–Fundus waves were longer (Fig. 4S) and higher magnitude (Fig. 4O) and power (Fig. 4W) in the healthy participants than in those with endometriosis. Conversely, the Fundus–Cervix waves in the peri-ovulatory phase were longer duration (Fig. 4T) and higher magnitude (Fig. 4P) in the participants with endometriosis than in the healthy participants.

### Peristalsis wave direction during ovulation correlates with dominant follicle laterality

**Figure 5:**
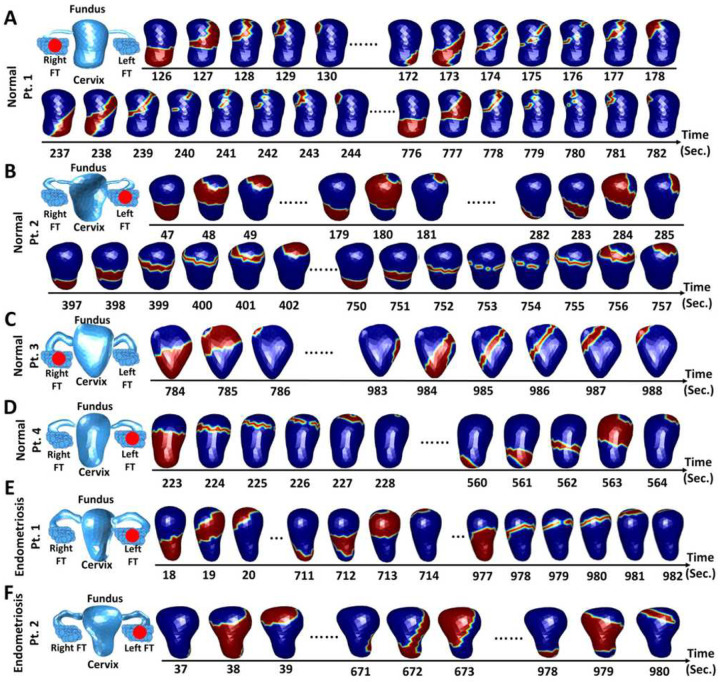
Representative asymmetric uterine peristalsis patterns in healthy participants with the normal menstrual cycle (A-D) and endometriosis patients (E-F) during the ovulatory phase. In each panel, anatomical uterus geometry with fallopian tubes was segmented from the T1-weighted and T2-weighted MRI images. Red dots indicate the ovary with the dominant follicle. (A, C) Normal patients 1 and 3 have left-dominant follicles and left-sided asymmetric uterine peristalsis propagation. (B, D) Normal participants 2 and 4 have right-dominant follicles and right-sided asymmetric uterine peristalsis propagation. (E, F) Endometriosis patients with left dominant follicles and right-sided asymmetric uterine peristalsis propagation. Patient numbers correspond with data in Table 1

Finally, we found that Cervix–Fundus peristalsis waves during the peri-ovulatory phase tend to move preferentially toward one fallopian tube. In nine of the healthy participants and two of the participants with endometriosis, we were able to determine which ovary had a dominant follicle by clinical TVUS and then observe whether the peristalsis propagated in the direction of the dominant follicle. Fig. 5A shows an example of UPI from a healthy participant with a dominant follicle in the right ovary. In this patient, 5 of 8 Cervix–Fundus peristalsis episodes moved toward the right ovary. The other 3 waves showed no preferential direction. Fig. 5B–D show additional examples of healthy participants in which peristalsis patterns propagated toward the ovary with the dominant follicle. Fig. 5E shows an example of a participant with endometriosis and a dominant follicle in the left ovary. In this participant, 4 out of 5 peristalsis cycles progressed toward the right fallopian tube and 1 progressed toward the left fallopian tube. Fig. 5F shows a second participant with endometriosis and a dominant follicle in the left ovary. In this participant, 6 out of 13 Cervix–Fundus peristalsis waves moved in the direction of the right fallopian tube, while none moved toward the left fallopian tube.

In the eight healthy participants for whom we had TVUS imaging demonstrating the dominant follicle, peristalsis waves during the ovulatory phase more often moved toward the side with the dominant follicle than toward the side with no dominant follicle. In two participants with endometriosis for whom we had data regarding the dominant follicle, the peristalsis waves during the ovulatory phase more often moved toward the side without the dominant follicle than toward the side with the dominant follicle (Table 1).

## Discussion

The UPI imaging data presented herein suggest that UPI can provide objective and quantitative measures of uterine peristalsis throughout the human menstrual cycle. Additionally, we developed novel indices to quantitatively characterize uterine peristalsis patterns automatically. Finally, we used UPI to provide evidence that uterine peristalsis patterns differ in women with normal anatomy and menstrual cycles and in women with endometriosis.

In the normal participants, the predominant peristalsis pattern in menses was Fundus-Cervix. This pattern has been seen by others and postulated to facilitate the expulsion of blood and endometrial tissue while protecting against ascending pathogens^36^. In the peri-ovulatory phase, the predominant peristalsis pattern was Cervix-Fundus. Kunz et al. used serial HSSG to follow labeled macrospheres the size of sperm and observed that they were transported from the cervix into the uterus and fallopian tubes^37^, suggesting that the Cervix-Fundus peristalsis pattern facilitates the transport of sperm toward the oocyte. We observed no predominant pattern in the proliferative and secretory phases. The duration and magnitude of contractions differed in each phase. The rise in oxytocin and estrogen in the follicular phase may explain why the magnitude of the peristalsis pattern is increased during menses^1,38,39^. After ovulation, during the secretory phase, progesterone (a known muscle relaxant) contributes to the decrease in the magnitude of peristalsis by antagonizing the estrogen and oxytocin receptors^40^.

Endometriosis has long been hypothesized to be caused by retrograde menstruation^13,41–46^. However, as all reproductive-age women have some amount of retrograde menstruation, it is unclear why only 10–15% of females would develop endometriosis^42,45,47–49^. We found that all healthy participants had at least some Cervix–Fundus peristalses, which could cause retrograde menstruation. Our data suggested that Cervix-Fundus peristalsis waves were less frequent and weaker than the Fundus–Cervix waves in subjects without endometriosis. Therefore, the strong and frequent Fundus-Cervix waves may have effectively expelled blood vaginally and left a small amount of blood in the uterine cavity. Although part of the blood could still be transported retrogradely to the peritoneal space by the weak Cervix–Fundus waves, the level may not be sufficient to cause endometriosis in healthy people. On the contrary, in participants with endometriosis, a higher percentage of waves were Cervix–Fundus, and these were stronger and had longer durations than the Cervix–Fundus waves in normal patients. More importantly, in healthy subjects, the Fundus-Cervix peristalsis waves were less frequent and weaker in endometriosis patients than the Fundus-Cervix peristalsis waves, which impair normal expulsion and leave more blood in the uterine cavity. Therefore, retrograde menstruation is more likely to push much more blood and tissue into the peritoneal space in women with endometriosis than in women without endometriosis^8,12,50,51^. Our work suggests that a comprehensive evaluation of 4D uterine peristalsis direction distribution, frequency, magnitude, duration, and power during the menses phase could be used to stratify the risk of developing endometriosis and assess the severity of endometriosis.

Our data may also provide clues to infertility in women with endometriosis. In healthy participants during the peri-ovulatory phase, uterine peristalsis waves most frequently traveled Cervix–Fundus, with most peristalsis waves traveling toward the dominant follicle. These patterns could assist sperm in transit to ensure interaction with an oocyte. Conversely, in participants with endometriosis during the peri-ovulatory phase, uterine peristalsis waves most frequently traveled Fundus–Cervix, and those that traveled Cervix–Fundus traveled toward the ovary without a dominant follicle more often than toward the ovary with a dominant follicle. These patterns could limit the number of spermatozoa that reach the oocyte^20,21,52,53^.

The UPI system potentially has a wide range of possible clinical research and therapeutic applications. Based on the initial work presented in this work, UPI can be used to further establish reference baseline parameters of uterine peristalsis in normal menstrual cycles. These baseline values could be used to create a composite score to identify patients with abnormal gynecological conditions such as endometriosis, ovulatory dysfunction, abnormal uterine bleeding, or amenorrhea. Additionally, UPI could be used to correlate the dominant follicle with uterine peristalsis direction in the peri-ovulatory phase and to develop a predictive biomarker for successful natural conception. With the detailed 4D electrical activation patterns imaged by UPI, we can longitudinally evaluate the treatment effects of various clinical interventions and optimize the treatment plan for an individual patient. In addition, UPI may facilitate the development of nonpharmaceutical treatments to electrically correct abnormal uterine peristalsis underlying various gynecological conditions, such as endometriosis, etc., using electronic devices similar to cardiac pacemakers.

UPI has several advantages over other modalities used to image uterine peristalsis. First, UPI is noninvasive, which is optimal for long-duration uterine monitoring. Additionally, modalities using invasive monitoring may iatrogenically cause non-physiologic perturbations of peristalsis. Second, UPI provides high spatial-temporal resolution information, including the initiation sites, direction, frequency, and duration of uterine peristalsis waves. Third, UPI provides 4D data that considers the individual’s unique uterine anatomy in both space and time domains. Fourth, UPI software allows automatic, objective, and real-time electrophysiological quantification of uterine peristalsis. Future work will focus on developing a portable, low-cost, wearable UPI system to enable larger UPI studies. To make UPI more accessible to patients, we will replace the current short anatomical MRI scan with a low-cost ultrasound measurement to generate the patient-specific body-uterus geometry. Wearable, low-cost, printed electrodes^54,55^ will also be integrated into the UPI system to minimize the costs.

## Materials and Methods

### Study design and participants

This study was performed in the Division of Reproductive Endocrinology & Infertility at Washington University School of Medicine. This study was approved by the Washington University Institutional Review Board, and all participants signed informed consent documents. Participants were included if they were female at birth, between the ages of 18 and 37 years. Normal participants were included if they had regular, predictable menstrual cycles every 24–35 days. Participants with endometriosis were included if they had surgically confirmed endometriosis. Potential participants were excluded if they were post-menopausal, pregnant, or breastfeeding; had a uterine anomaly; had exposure to medications known to affect uterine contractility (e.g., magnesium, opioids, beta antagonists, nifedipine); were non-English speaking; had abdominal circumference > 55 cm; or had MRI contraindications (pacemaker, metal implants, etc.). Potential participants for the normal group were excluded if they had documented or self-reported histories of infertility, ovulatory dysfunction, or endometriosis. Potential participants for the endometriosis group were excluded if they were currently using female birth control. Seventeen out of them finished the longitudinal data acquisition and MRI study. Participants with regular menstrual cycles and five patients with endometriosis were enrolled in this study. Demographics and obstetric and gynecologic history of enrolled participants are shown in Supplemental Table 1. Each participant was imaged with the UPI system four times during one menstrual cycle, once during menses, early proliferative, late proliferative (peri-ovulatory), and secretory phases. Blood was collected at each visit to measure concentrations of the hormones estradiol, progesterone, and testosterone to confirm the menstrual phase.

### Definition of menstrual phases

Patients were determined to be in one of four menstrual phases (menses, early proliferative, late proliferative, and secretory) by using a combination of patient-reported bleeding, cycle length, ultrasound findings, ovulation predictor kit (Clearblue, Geneva, Switzerland) results, and hormonal measurements. Serum blood (5–10 ml) was collected and sent to the Core Laboratory for Clinical Studies at Washington University in St. Louis to measure concentrations of the hormones (estradiol, progesterone, and testosterone). The menses phase was assigned when a patient-reported bleeding. The early proliferative phase was assigned after the patient had stopped bleeding, ultrasound demonstrated early follicular activity (largest follicle size <16 mm), serum estradiol <200 pg/ml, and serum progesterone <3 ng/ml. The late proliferative (peri-ovulatory) phase was defined by a positive result on an ovulation predictor kit, serum estradiol >200 pg/ml, serum progesterone <3 ng/ml, and/or a dominant follicle on ultrasound (⩾16mm). The secretory phase was assigned when serum progesterone was >3 ng/ml.

### Uterine peristalsis imaging (UPI) procedure

First, a woman underwent a one-time, fast, anatomical (T2W sequence) 3T Siemens Prisma MRI scan (~10 mins) to acquire the patient-specific uterus-body surface geometry while wearing up to 8 patches containing up to 128 MRI-compatible fiducial markers around the abdomen and lower back (Fig. 1A). Uterus and body geometry were generated (Fig. 1 B&C). Second, after the MRI scan, customized BioSemi pin-type electrode patches were applied to the same locations on the body surface as the MRI fiducial markers. An ADC box was used to record the body surface electrical signals (Fig. 1D&E) for 20 minutes. The body surface electrical signals were processed with a band-pass filter (0.01–0.1 Hz)^25,34,35^ to generate wave electrical signals (peristalsis waves) over the entire abdomen surface (Fig. 1F). Third, the participant underwent another 10-minute electrical recording while simultaneously undergoing transvaginal ultrasound (TVUS). TVUS scans of the uterus were performed by the sonographer holding the transducer probe while the patient was lying in a lithotomy position, and TVUS clips were recorded on a GE Voluson S8 ultrasound machine. The duration of each clip was 20 seconds on average, and 30–35 clips were acquired in total. A registered sonographer independently (without knowledge of the UPI results) examined the TVUS recordings to determine the uterine peristalsis direction.

### Inverse computation in UPI

With the electro-quasi-static assumption of the bioelectric field, the inverse computation combines the patient-specific uterus-abdomen surface and electrical potentials measured on the abdominal surface to reconstruct the potential distribution over the entire 3D uterine surface. We assume that the medium is homogeneous between the uterine surface and abdominal surface without any primary electrical source. Then, the inverse problem could be mathematically described by the Cauchy problem for Laplace’s equation (1) with boundary conditions (2,3) on the abdominal surface.


(1) $$\nabla^{2}\phi (x)=0$$

Dirichlet (2) and Neumann (3) conditions for the abdominal surface potentials are:

(2) $$\phi (x)=\phi_{A(x)},x\in \Gamma_{A}$$

(3) $$\frac{\partial \phi (x)}{\partial n}=0,x\in \Gamma_{A}$$

Here, ***n*** is the normal vector on the abdominal surface at location *x* and Γ_*A*_ represents abdominal surface. *ϕ*_*A*(*x*)_ is the potential measured on the abdominal surface and *ϕ*(*x*) is the potential on the uterine surface.

As a mesh-free method robust to noise, a method of fundamental solutions (MFS)^56^ was deployed to discretize the Laplace’s equation and boundary conditions, which is accurate for solving the bioelectric field inverse problem in both electrocardiographic imaging (ECGI)^56^ and electromyometrial imaging(EMMI)^30,32,33^ systems. This problem cannot be solved directly as it is an ill-posed inverse problem. Therefore, Tikhonov-based inverse computation with a fixed regularization value of 0.01 was used to obtain the solution.


(4) $$\Phi_{A}=A\;\Phi_{U}$$

Here, Φ_*A*_ is a M * T matrix of measuring surface potentials, Φ_*U*_ is a N * T matrix of uterine surface potentials, where M is the number of measuring electrodes applied on the abdominal surface and N is the number of discrete points on the uterine surface, and T is the number of recording time points. ***A*** is a M * N linear transform matrix encoding the relationship between abdominal surface potential Φ_*A*_ and uterine surface potential Φ_*U*_.

### UPI data processing

The inverse computation described above was employed to compute the uterine surface electrical signals (Fig. 1 G&H) on the three-dimensional uterine surface. The times when the uterine surface electrical signals at various uterine surface areas reached the steepest negative slope^57–61^ were extracted and defined as electrical activation times at those uterine areas during peristalsis waves (red dots in Fig. 1 G&H). During each peristalsis wave, sequential time frames were generated as the activation sequences (Fig. 1I) to reflect the detailed 4D spatial-temporal activation patterns of the uterine peristalsis. Within each time frame, the red region indicated the electrically activated myometrium areas currently experiencing peristalsis, and the blue region indicated the inactive areas of the uterus. The isochrone map was generated as a color-coded 3D map to summarize the electrical activation sequence (Fig. 1J). In the isochrone map, warm and cool colors denote regions of the uterus that activated early and late, respectively, during the peristalsis wave. The UPI isochrone maps contained rich spatial-temporal information of uterine activation, including the activation and termination sites, propagation direction, and duration. In addition, uterine potential maps were generated to reflect the 4D electrical potential distribution during peristalsis waves: 1D electrical signals (Fig. 1 G&H) over the entire 3D uterine surface (Fig. 1K). The distributions of uterine peristalsis propagation direction, initiation, and termination sites (Fig. 1L) were automatically calculated as the number of peristalsis waves with a specific propagation direction (Fundus-Cervix, Cervix-Fundus or other), initiation, and termination site (cervical, fundal or other regions) divided by the total number of peristalsis waves in the 20-minute electrical mapping session, respectively.

### Electrophysiological characterization and quantification of human uterine peristalsis

Five UPI electrophysiological indices were defined to qualitatively and quantitatively describe uterine peristalsis patterns. First, the propagation direction was determined from the uterine peristalsis activation maps. Uterine peristalsis directions were classified into three categories: Fundus-Cervix, Cervix-Fundus, and others including Anterior-Posterior, Posterior-Anterior, Left-Right, and Right-Left. Second, the initiation and termination sites were defined as the region experiencing the earliest and latest activation during uterine peristalsis. The initiation and termination sites were identified on the isochrone maps and were classified into three categories: Cervical region, Fundal region, and Other regions. Third, the duration (Sec.) was defined as the duration of a complete peristalsis wave measured in the isochrone map of the uterine peristalsis wave. A small fraction of uterine peristalsis waves only involve the partial activation of the uterus and has a relatively shorter duration. Fourth, uterine peristalsis magnitude (mV) was defined as the average peak amplitude of electrical potential over the uterine region experiencing activation during the entire peristalsis wave. Finally, uterine peristalsis power (mV*sec) was defined as the product of magnitude and duration for each uterine peristalsis.

### Definition of cervix-fundus uterine peristalsis wave laterality

The distance between the latest fundus-activated uterine site and the left fallopian tube insertion site was defined as *d*_*left*_,, the distance between the latest fundus-activated uterine site and the right fallopian tube insertion site was defined as *d*_*right*_,,. The ratio between these two distances was defined as $R=\frac{d_{\text{left}}}{d_{\text{right}}}$. If *R* < 0.8, the cervix-fundus uterine peristalsis was left dominant; if *R* > 1.25, the cervix-fundus uterine peristalsis was right dominant; if 0.8 < *R* < 1.25, the cervix-fundus uterine peristalsis was middle dominant with no side preference.
